# Supplementary material for: GABAA Receptor Ligands Often Interact with Binding Sites in the Transmembrane Domain and in the Extracellular Domain—Can the Promiscuity Code Be Cracked?
Source: Int J Mol Sci. 2020 Jan 3;21(1):334. doi: 10.3390/ijms21010334 (PMC6982053; doi:10.3390/ijms21010334)
Supplement: Supplementary file 1 [file ijms-21-00334-s001.pdf]

GABA$_A$ receptor ligands often interact with binding sites in the transmembrane domain and in the extracellular domain – can the promiscuity code be cracked?

Maria Teresa Iorio [1], Florian D. Vogel[2], Filip Koniuszewski[2], Petra Scholze[2], Sabah Rehman[2], Xenia Simeone[2], Michael Schnürch [1], Marko D. Mihovilovic [1] and Margot Ernst[2,*]

[1] TU Wien, Institute of Applied Synthetic Chemistry, Getreidemarkt 9/163-OC, 1060, Vienna, Austria;
[2] Medical University of Vienna, Center for Brain Research Spitalgasse 4, 1090 Vienna, Austria;

Supporting information

^{1}H NMR and ^{13}C NMR Spectrums of Novel Characterized Compounds

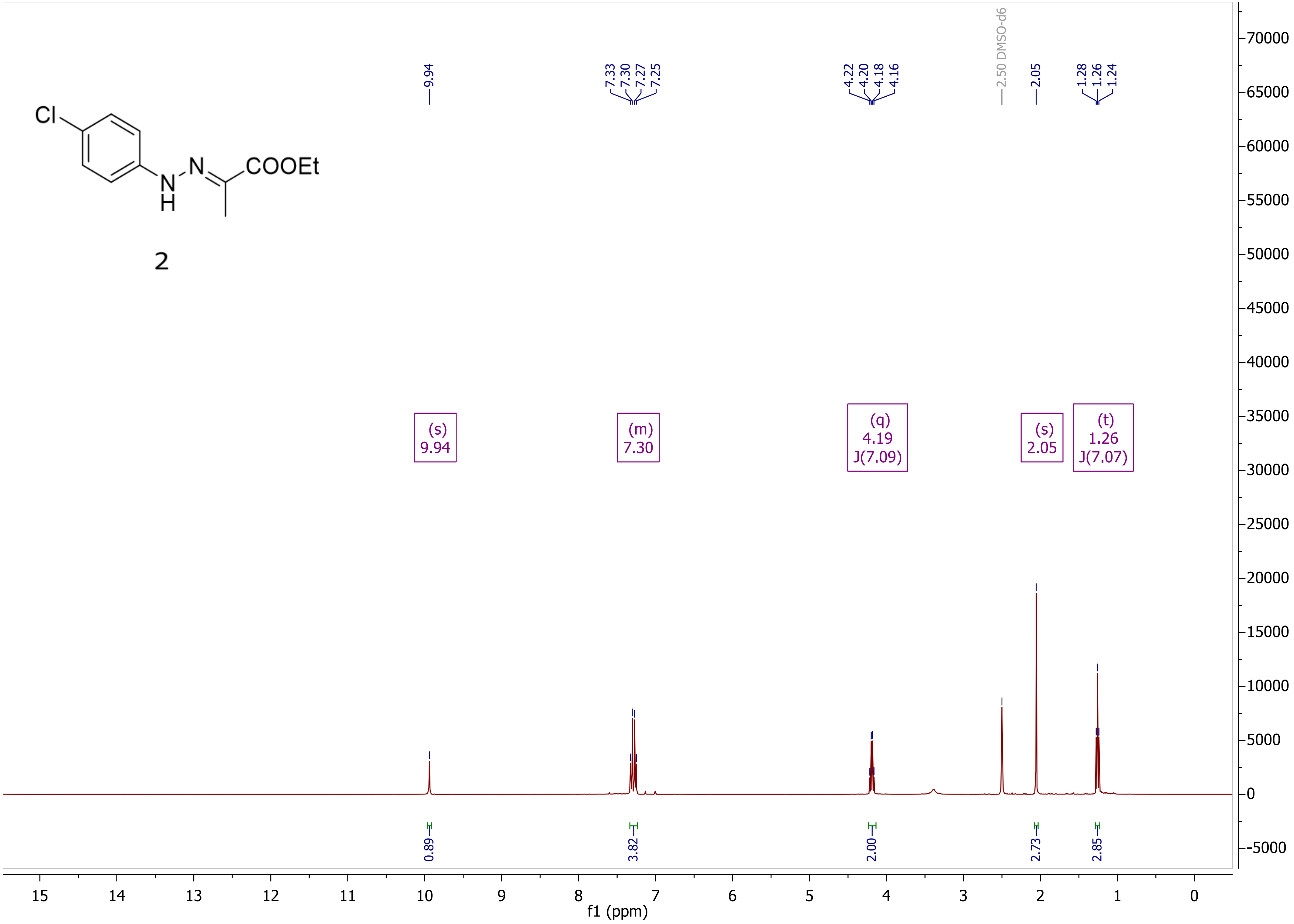

Cl
N
N
H
COOEt
2
9.94
7.33
7.30
7.27
7.25
4.22
4.20
4.18
4.16
2.50 DMSO-d6
2.05
1.28
1.26
1.24
(s) 9.94
(m) 7.30
(q) 4.19 J(7.09)
(s) 2.05
(t) 1.26 J(7.07)
0.89
3.82
2.00
2.73
2.85
15 14 13 12 11 10 9 8 7 6 5 4 3 2 1 0
f1 (ppm)
70000 65000 60000 55000 50000 45000 40000 35000 30000 25000 20000 15000 10000 5000 0 -5000

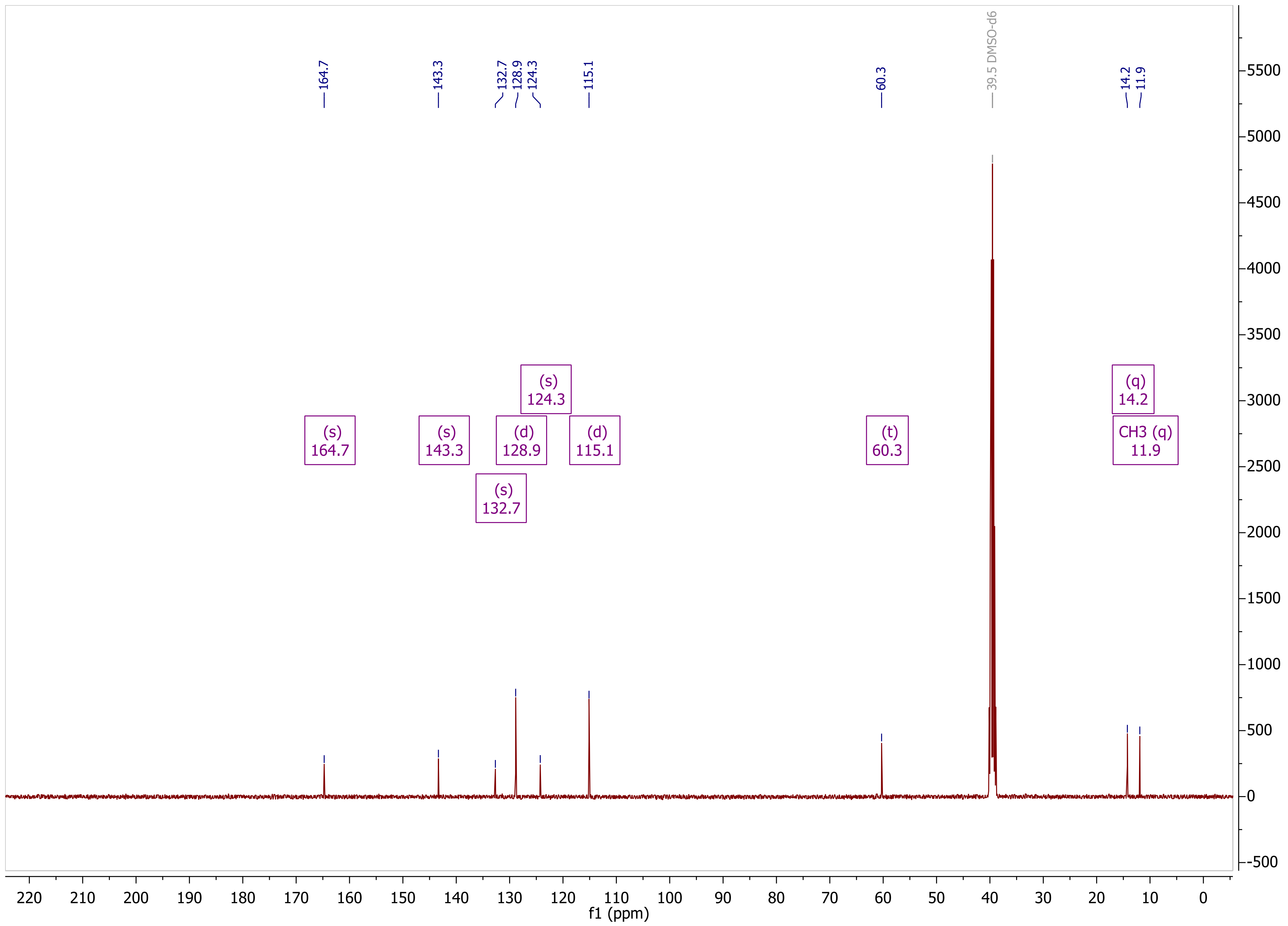

164.7
143.3
132.7
128.9
124.3
115.1
60.3
39.5 DMSO-d6
14.2
11.9
(s) 164.7
(s) 143.3
(s) 132.7
(d) 128.9
(s) 124.3
(d) 115.1
(t) 60.3
(q) 14.2
CH3 (q) 11.9
f1 (ppm)

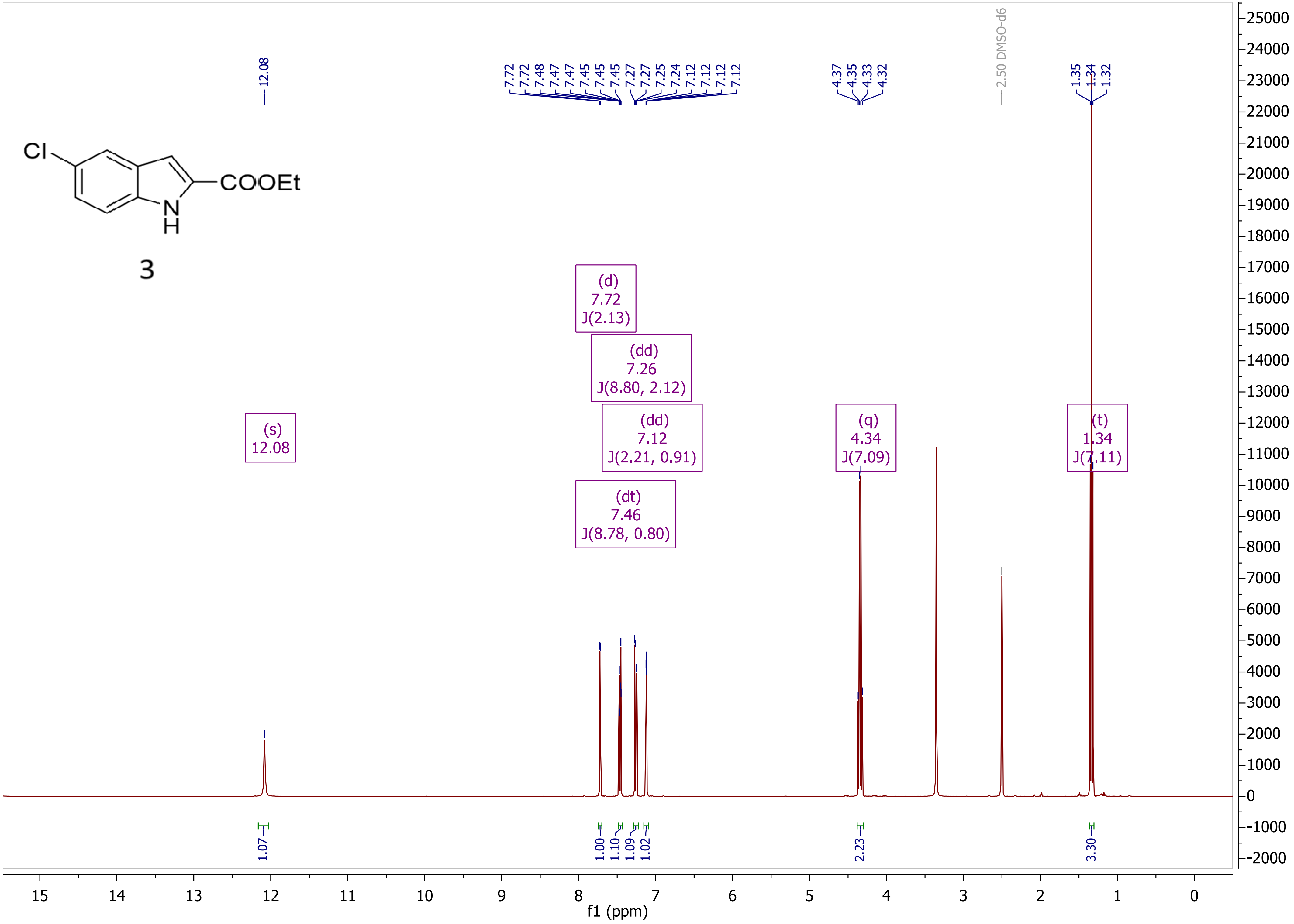

12.08
7.72 7.72 7.48 7.47 7.47 7.45 7.45 7.45 7.27 7.27 7.25 7.24 7.12 7.12 7.12 7.12
4.37 4.35 4.33 4.32
2.50 DMSO-d6
1.35 1.34 1.32
Cl
COOEt
N
H
3
(d) 7.72 J(2.13)
(dd) 7.26 J(8.80, 2.12)
(dd) 7.12 J(2.21, 0.91)
(dt) 7.46 J(8.78, 0.80)
(s) 12.08
(q) 4.34 J(7.09)
(t) 1.34 J(7.11)
1.07
1.00
1.10
1.09
1.02
2.23
3.30
f1 (ppm)

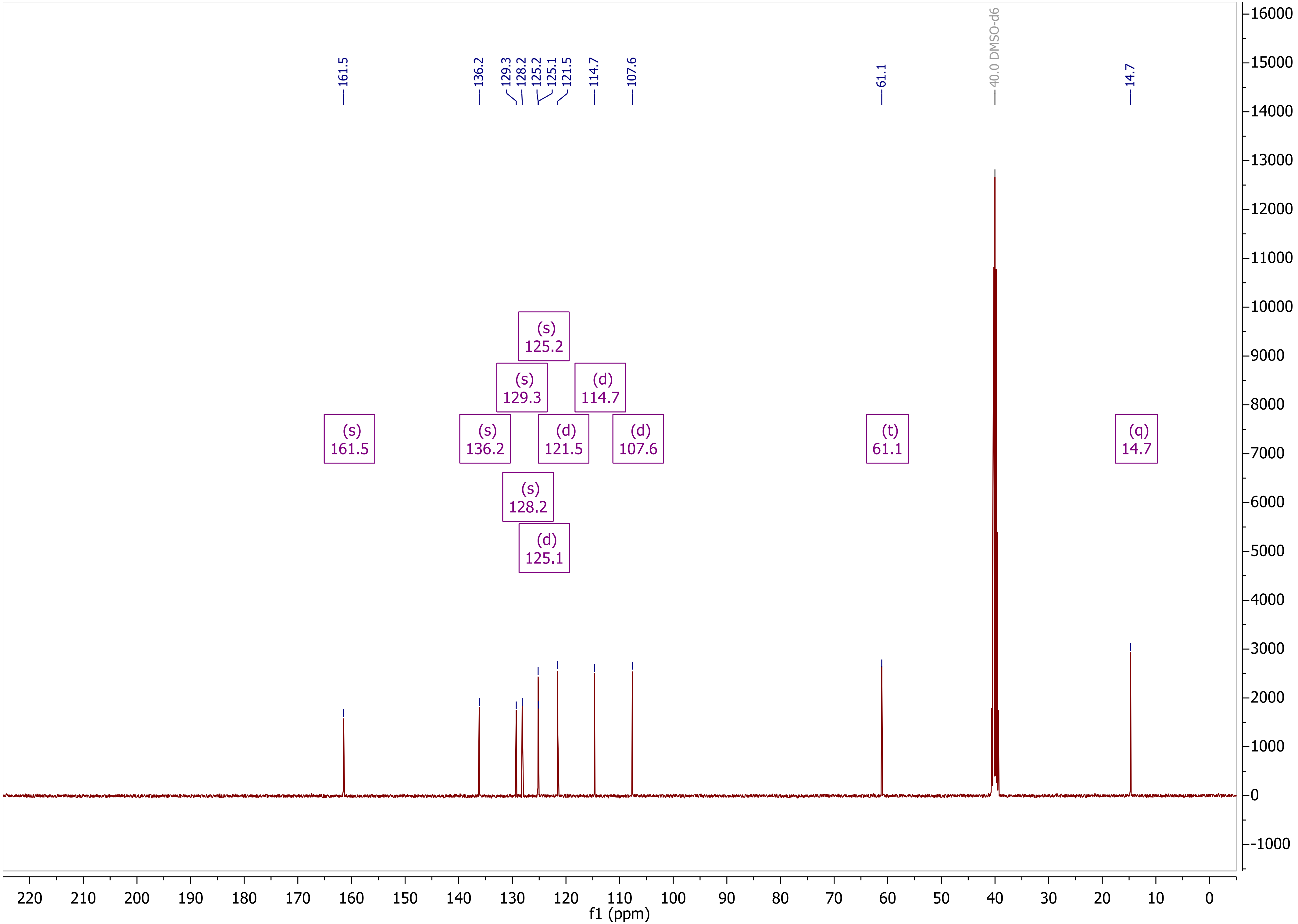

161.5
136.2
129.3
128.2
125.2
125.1
121.5
114.7
107.6
61.1
40.0 DMSO-d6
14.7
(s) 161.5
(s) 136.2
(s) 129.3
(s) 128.2
(s) 125.2
(d) 125.1
(d) 121.5
(d) 114.7
(d) 107.6
(t) 61.1
(q) 14.7
f1 (ppm)

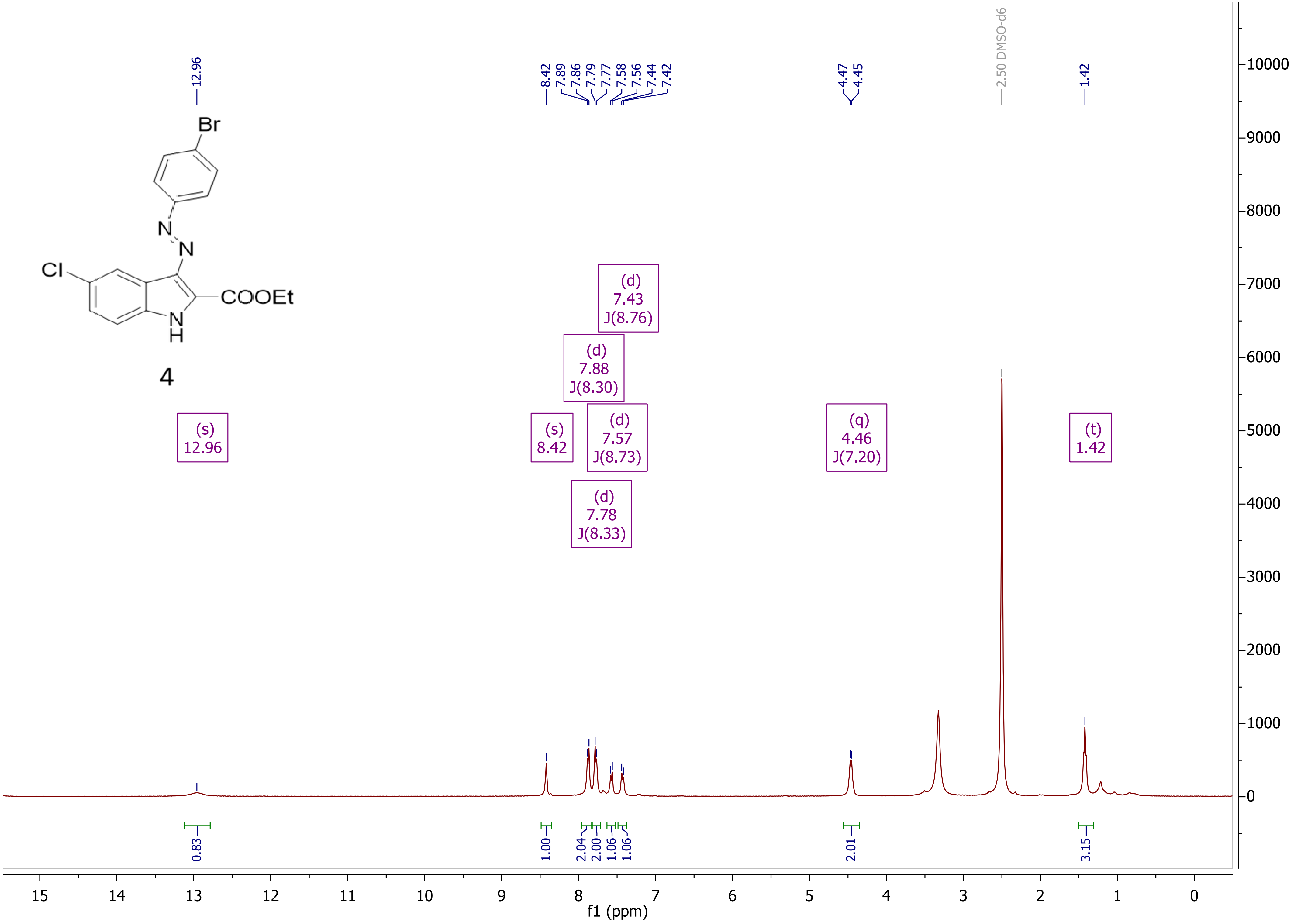

12.96
8.42
7.89
7.86
7.79
7.77
7.58
7.56
7.44
7.42
4.47
4.45
2.50 DMSO-d6
1.42
Br
N
N
Cl
COOEt
N
H
4
(d) 7.43 J(8.76)
(d) 7.88 J(8.30)
(s) 12.96
(s) 8.42
(d) 7.57 J(8.73)
(q) 4.46 J(7.20)
(t) 1.42
(d) 7.78 J(8.33)
0.83
1.00
2.04
2.00
1.06
1.06
2.01
3.15
15 14 13 12 11 10 9 8 7 6 5 4 3 2 1 0
f1 (ppm)
10000 9000 8000 7000 6000 5000 4000 3000 2000 1000 0

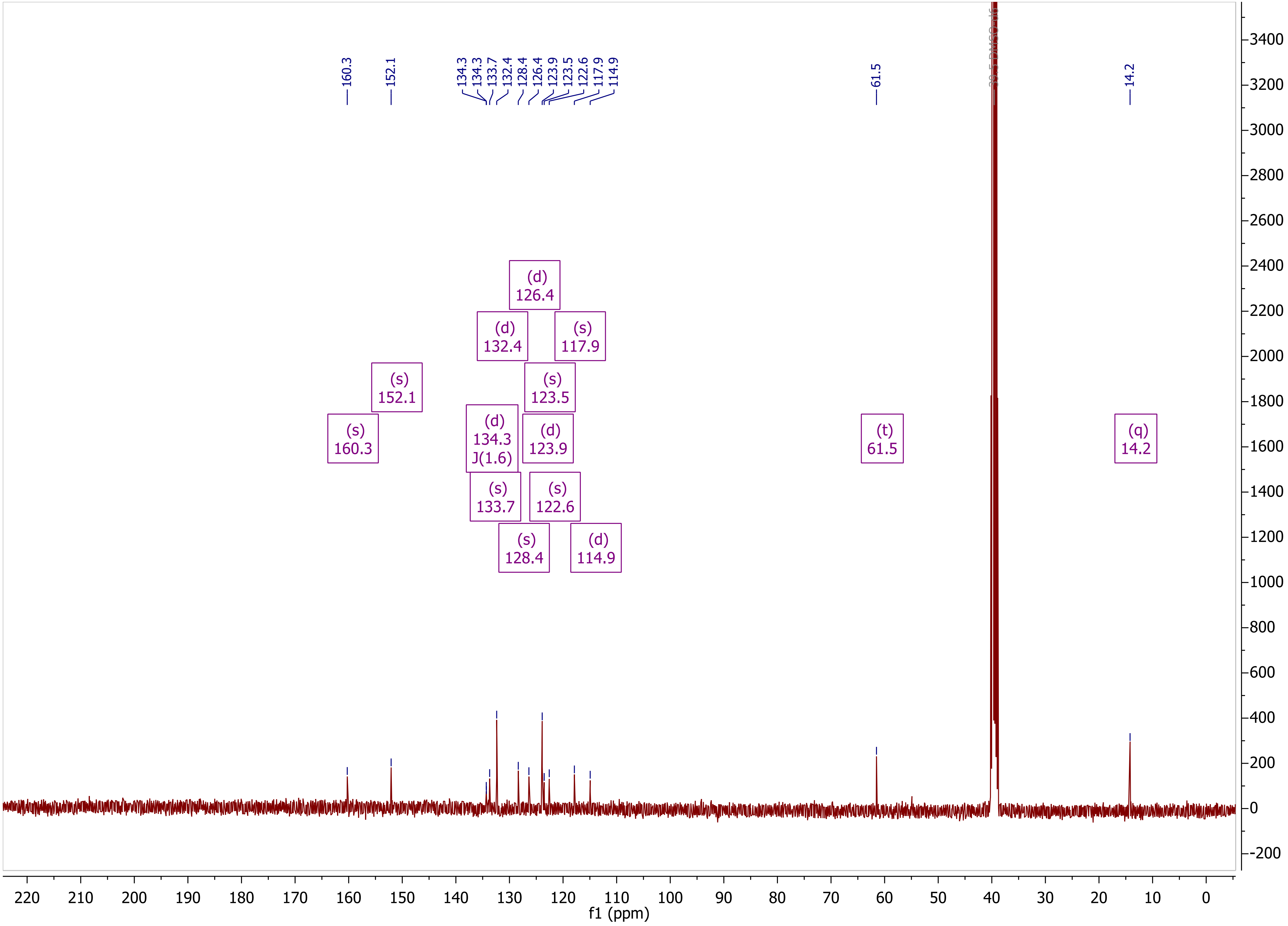

160.3
152.1
134.3
134.3
133.7
132.4
128.4
126.4
123.9
123.5
122.6
117.9
114.9
61.5
14.2
(d) 126.4
(d) 132.4
(s) 117.9
(s) 152.1
(s) 123.5
(s) 160.3
(d) 134.3 J(1.6)
(d) 123.9
(t) 61.5
(q) 14.2
(s) 133.7
(s) 122.6
(s) 128.4
(d) 114.9
f1 (ppm)

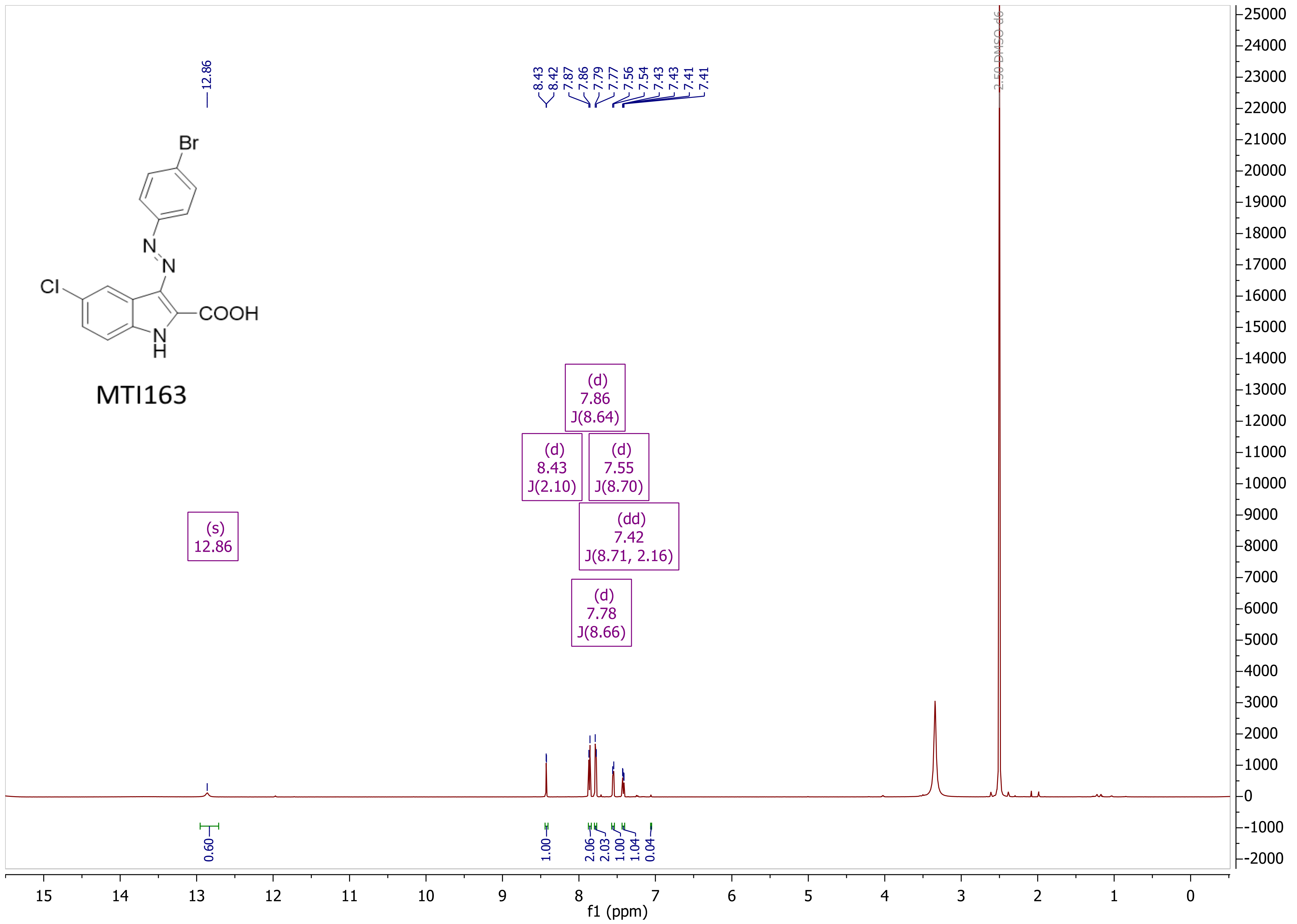

MTI163
Br
N
N
Cl
COOH
N
H
12.86
8.43
8.42
7.87
7.86
7.79
7.77
7.56
7.54
7.43
7.43
7.41
7.41
2.50 DMSO d6
(d) 7.86 J(8.64)
(d) 8.43 J(2.10)
(d) 7.55 J(8.70)
(s) 12.86
(dd) 7.42 J(8.71, 2.16)
(d) 7.78 J(8.66)
0.60
1.00
2.06
2.03
1.00
1.04
0.04
f1 (ppm)

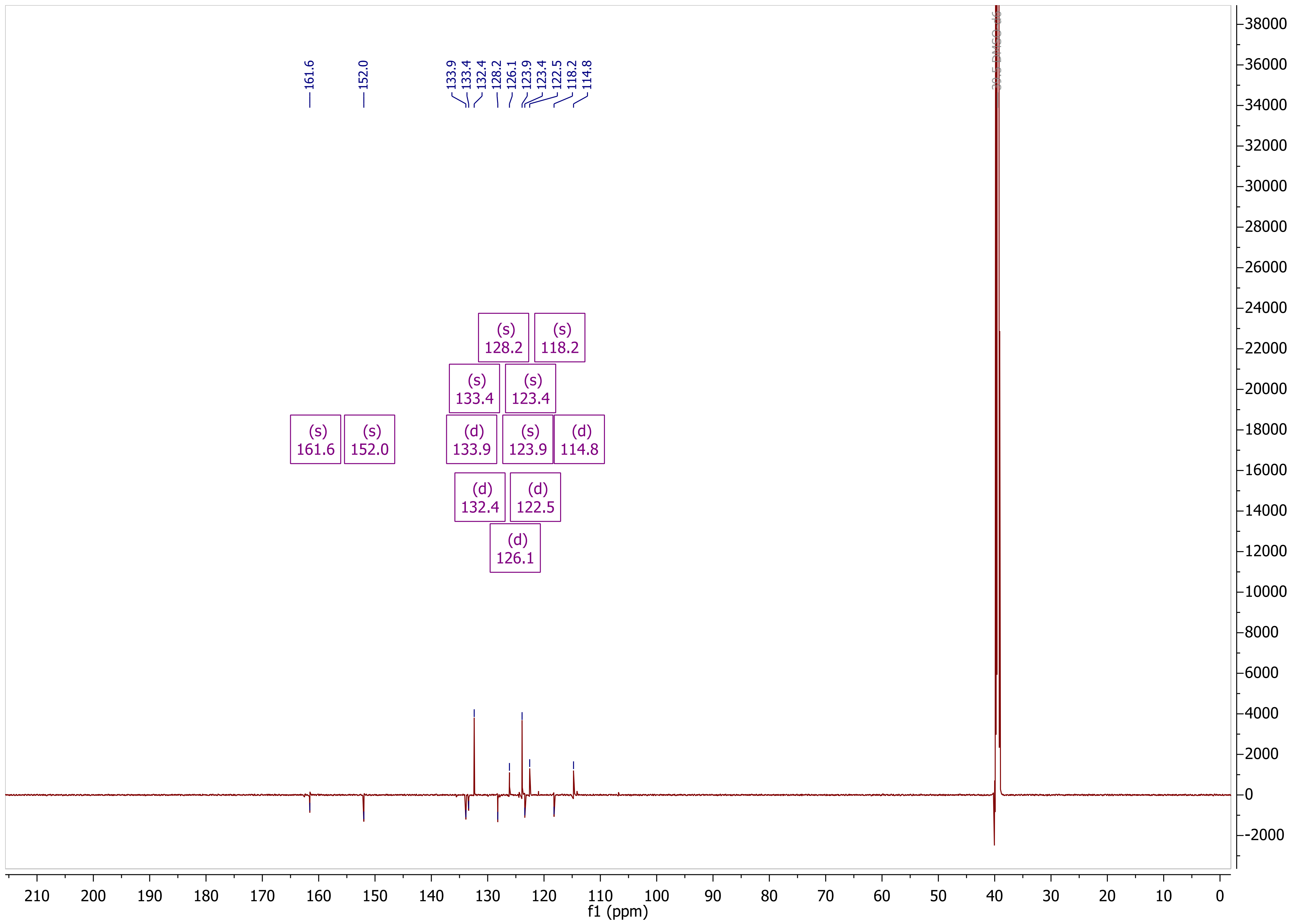
161.6
152.0
133.9
133.4
132.4
128.2
126.1
123.9
123.4
122.5
118.2
114.8
39.5 DMSO-d6
(s) 128.2
(s) 118.2
(s) 133.4
(s) 123.4
(s) 161.6
(s) 152.0
(d) 133.9
(s) 123.9
(d) 114.8
(d) 132.4
(d) 122.5
(d) 126.1
210 200 190 180 170 160 150 140 130 120 110 100 90 80 70 60 50 40 30 20 10 0
f1 (ppm)
38000 36000 34000 32000 30000 28000 26000 24000 22000 20000 18000 16000 14000 12000 10000 8000 6000 4000 2000 0 -2000

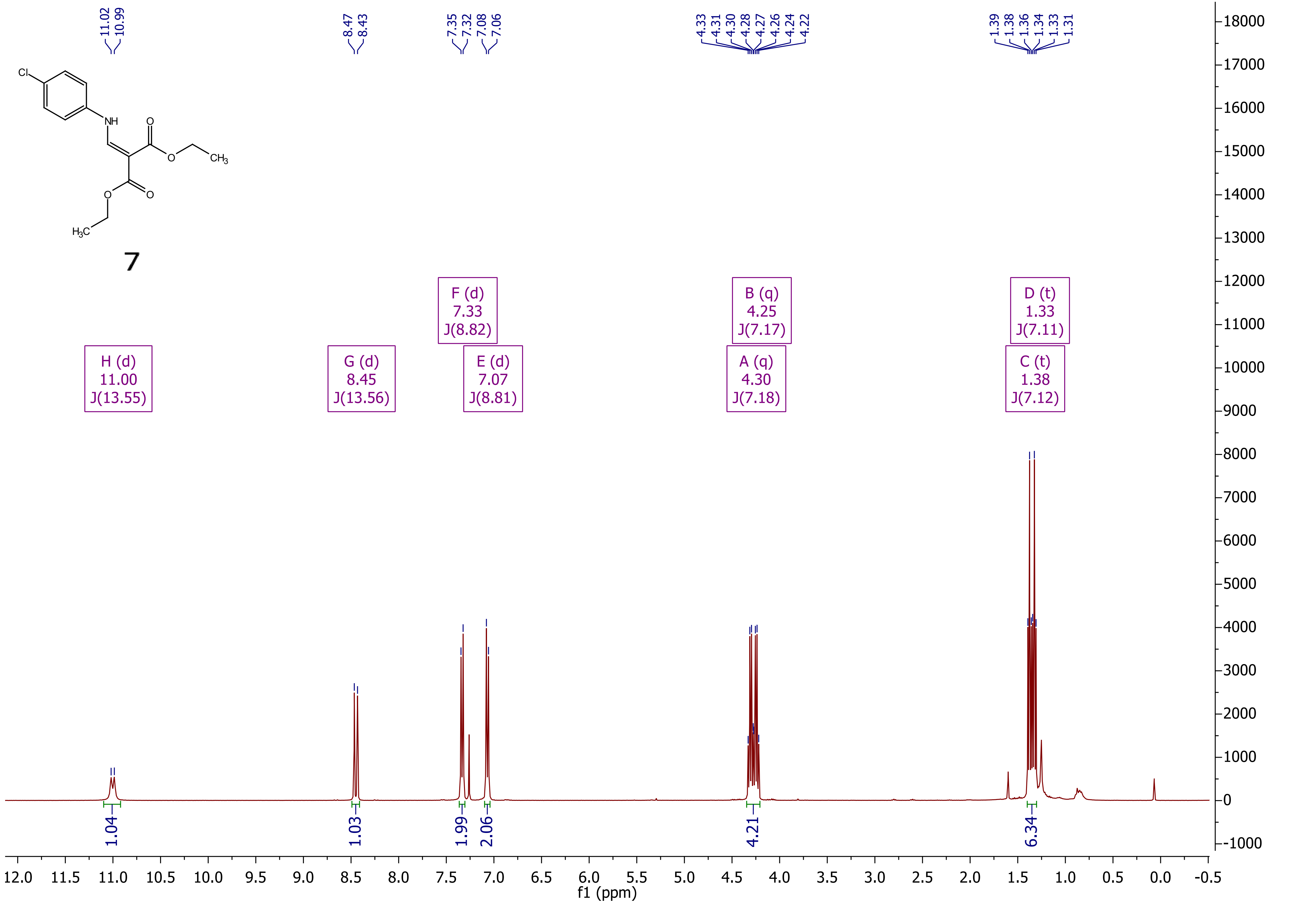

Cl
NH
O
O
CH3
O
O
H3C
7
11.02
10.99
8.47
8.43
7.35
7.32
7.08
7.06
4.33
4.31
4.30
4.28
4.27
4.26
4.24
4.22
1.39
1.38
1.36
1.34
1.33
1.31
F (d) 7.33 J(8.82)
B (q) 4.25 J(7.17)
D (t) 1.33 J(7.11)
H (d) 11.00 J(13.55)
G (d) 8.45 J(13.56)
E (d) 7.07 J(8.81)
A (q) 4.30 J(7.18)
C (t) 1.38 J(7.12)
1.04
1.03
1.99
2.06
4.21
6.34
f1 (ppm)

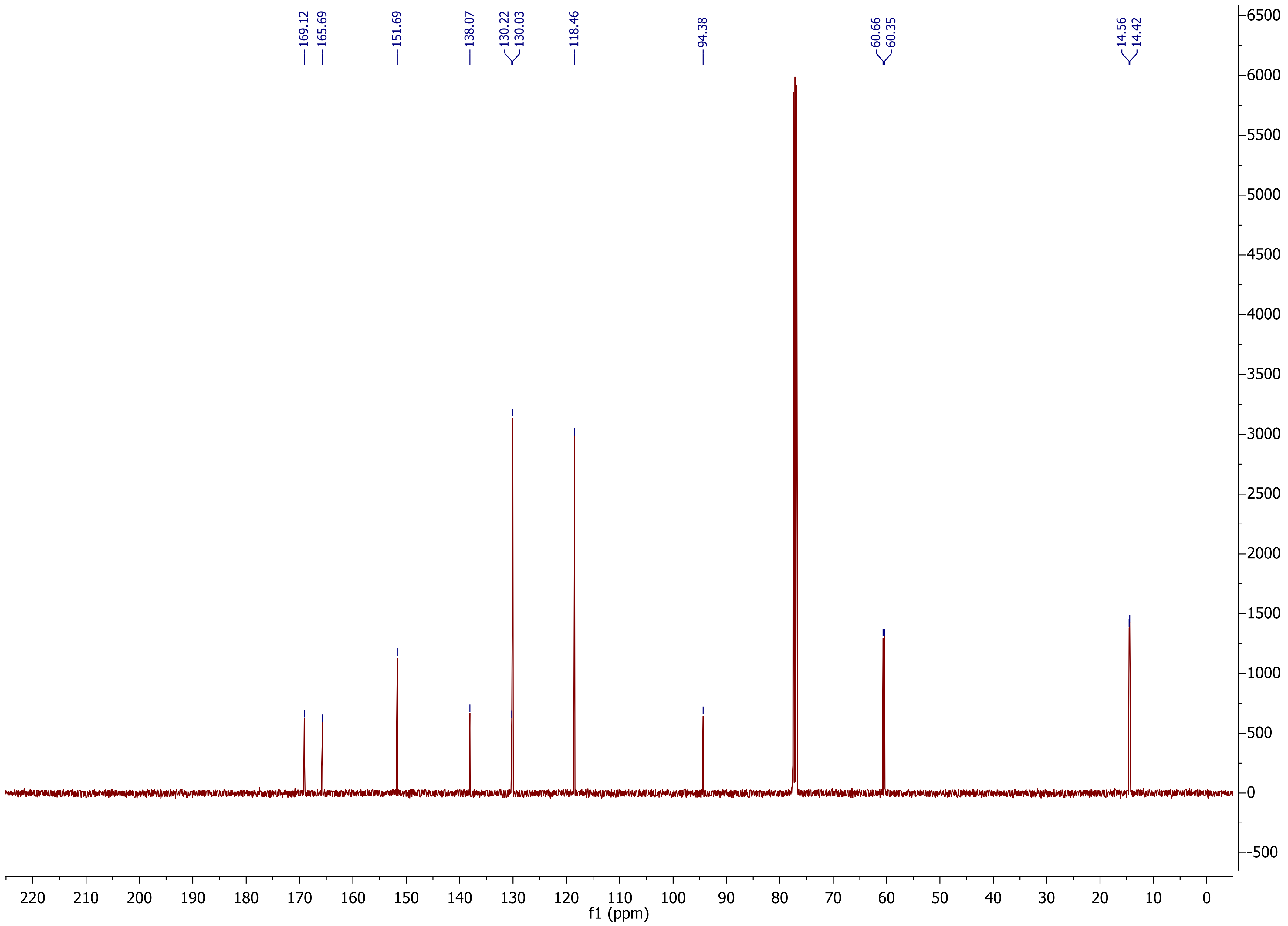
169.12
165.69
151.69
138.07
130.22
130.03
118.46
94.38
60.66
60.35
14.56
14.42
f1 (ppm)

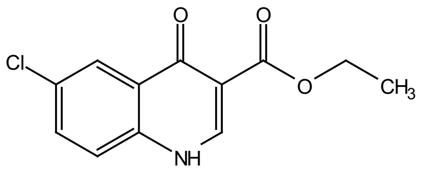

Cl
O
O
O
CH3
NH

8

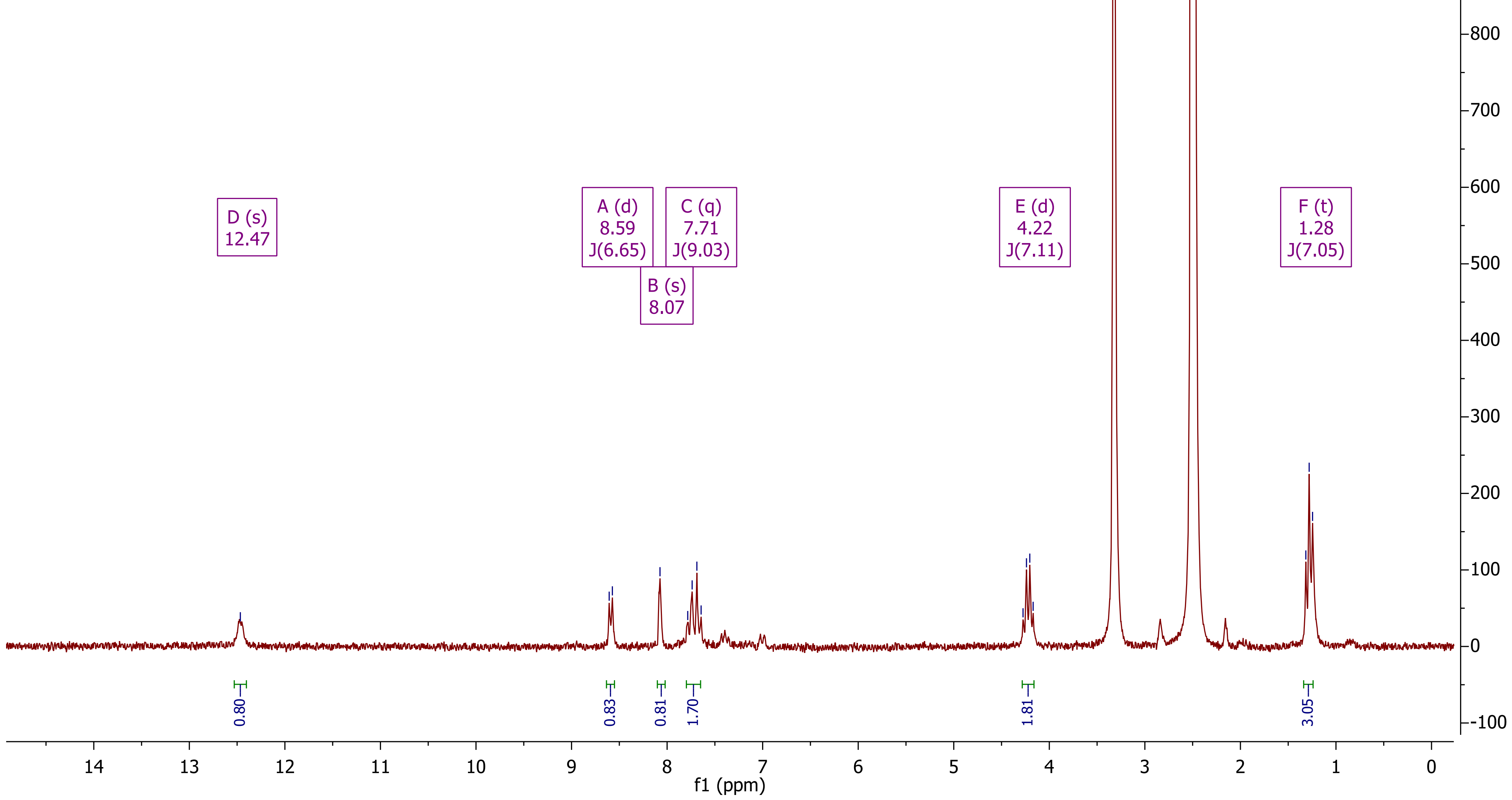

12.47
8.61
8.57
8.07
7.78
7.74
7.69
7.64
4.28
4.24
4.20
4.17
1.32
1.28
1.24
D (s)
12.47
A (d)
8.59
J(6.65)
C (q)
7.71
J(9.03)
B (s)
8.07
E (d)
4.22
J(7.11)
F (t)
1.28
J(7.05)
0.80
0.83
0.81
1.70
1.81
3.05
14
13
12
11
10
9
8
7
6
5
4
3
2
1
0
f1 (ppm)
1200
1100
1000
900
800
700
600
500
400
300
200
100
0
-100

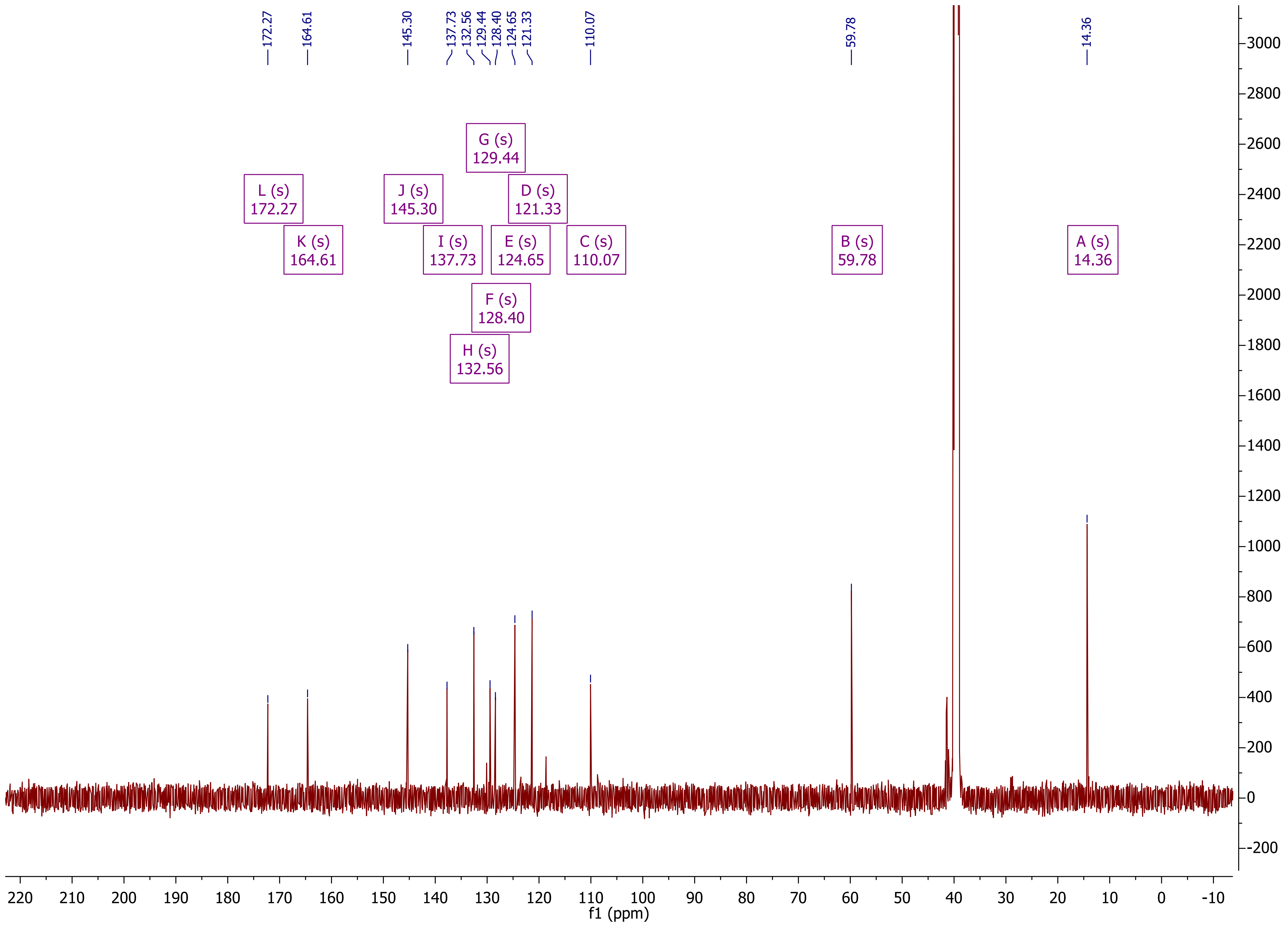

172.27
164.61
145.30
137.73
132.56
129.44
128.40
124.65
121.33
110.07
59.78
14.36
G (s) 129.44
L (s) 172.27
J (s) 145.30
D (s) 121.33
K (s) 164.61
I (s) 137.73
E (s) 124.65
C (s) 110.07
B (s) 59.78
A (s) 14.36
F (s) 128.40
H (s) 132.56
3000
2800
2600
2400
2200
2000
1800
1600
1400
1200
1000
800
600
400
200
0
-200
220 210 200 190 180 170 160 150 140 130 120 110 100 90 80 70 60 50 40 30 20 10 0 -10
f1 (ppm)

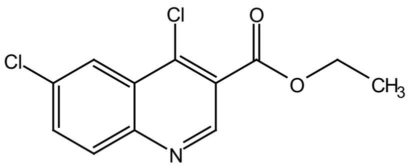
Cl
O
Cl
O
CH3
N

9

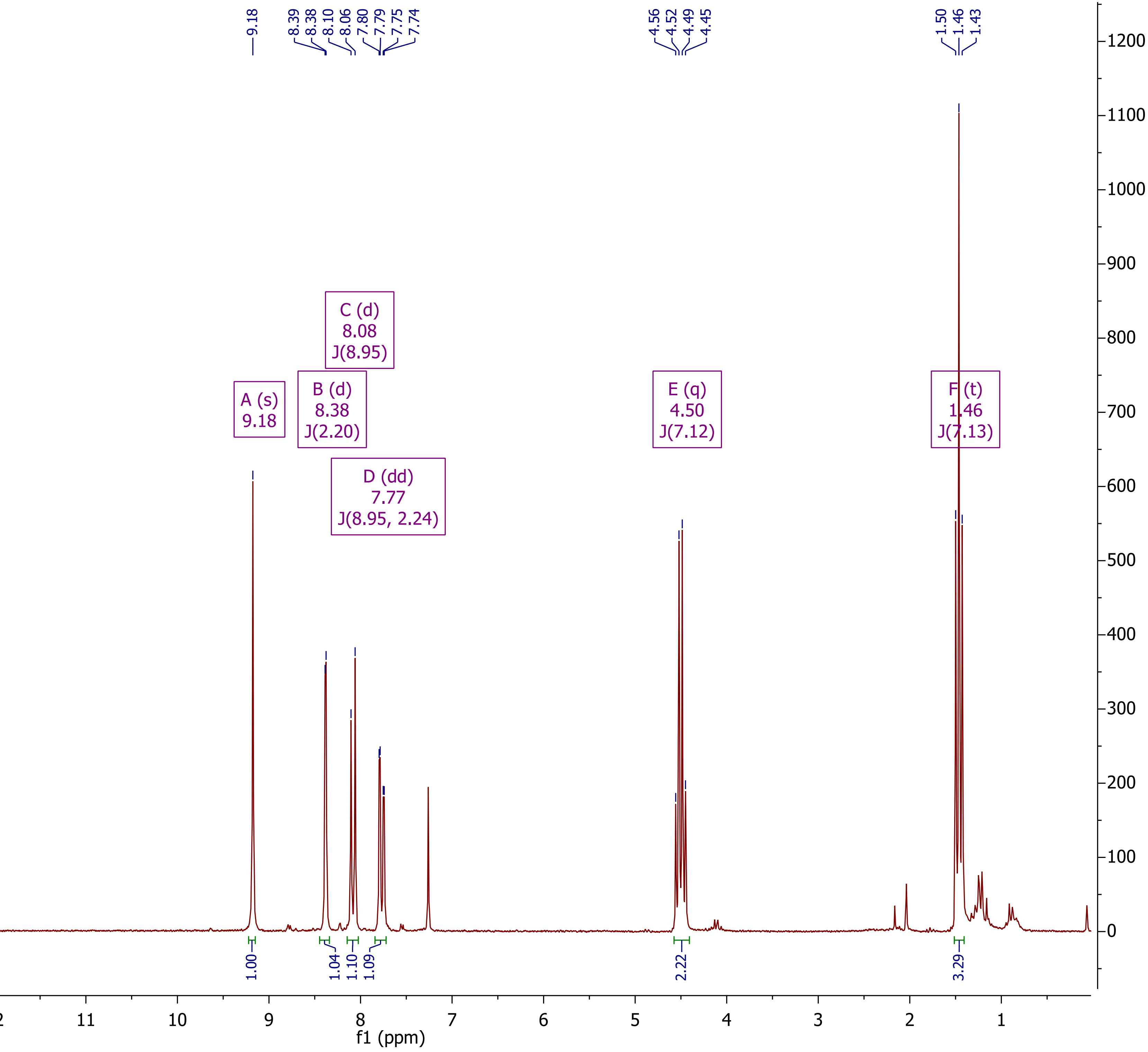
9.18
8.39
8.38
8.10
8.06
7.80
7.79
7.75
7.74
4.56
4.52
4.49
4.45
1.50
1.46
1.43
A (s)
9.18
B (d)
8.38
J(2.20)
C (d)
8.08
J(8.95)
D (dd)
7.77
J(8.95, 2.24)
E (q)
4.50
J(7.12)
F (t)
1.46
J(7.13)
1.00
1.04
1.10
1.09
2.22
3.29
15
14
13
12
11
10
9
8
7
6
5
4
3
2
1
f1 (ppm)
1200
1100
1000
900
800
700
600
500
400
300
200
100
0

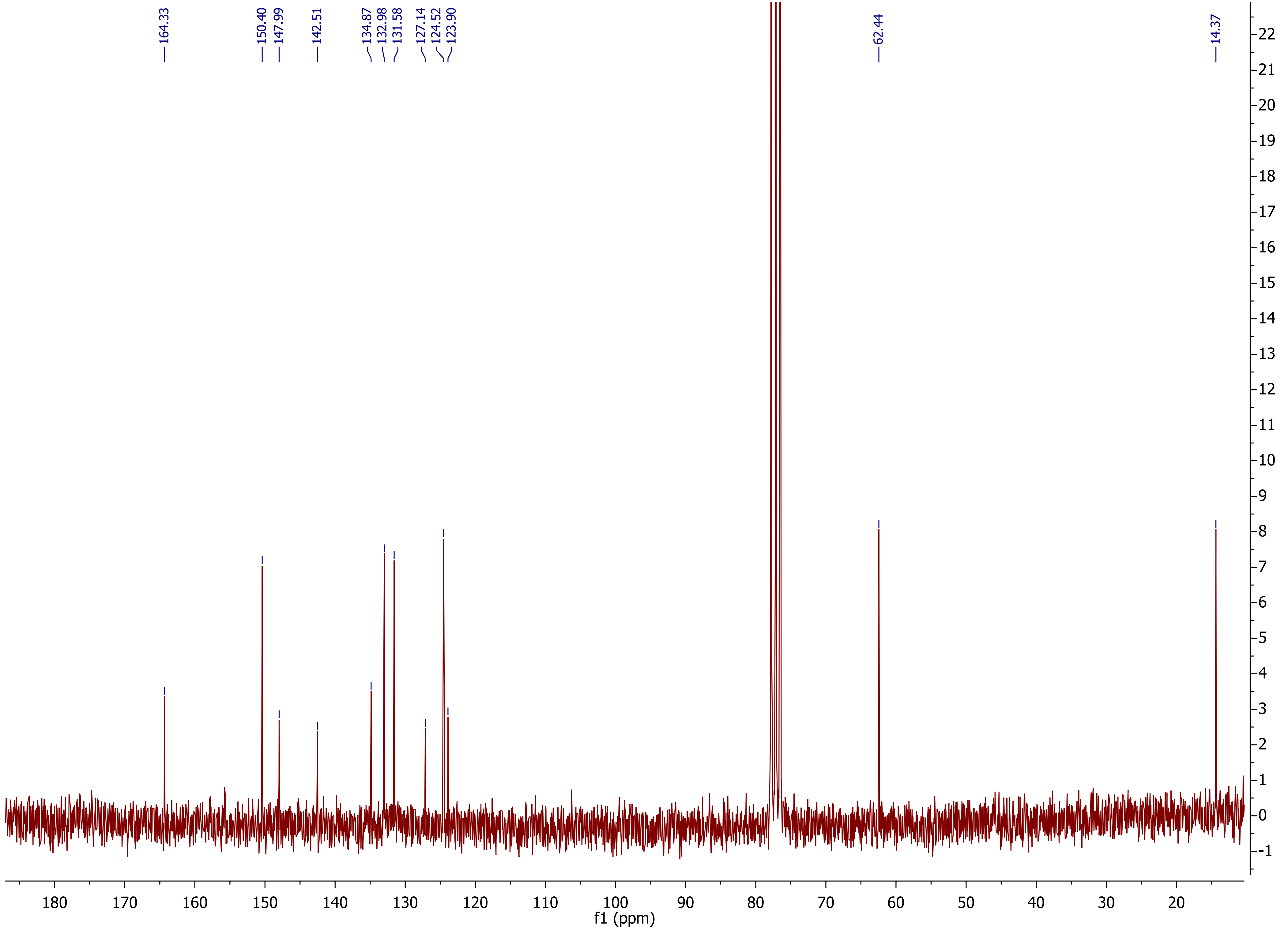

164.33
150.40
147.99
142.51
134.87
132.98
131.58
127.14
124.52
123.90
62.44
14.37
f1 (ppm)

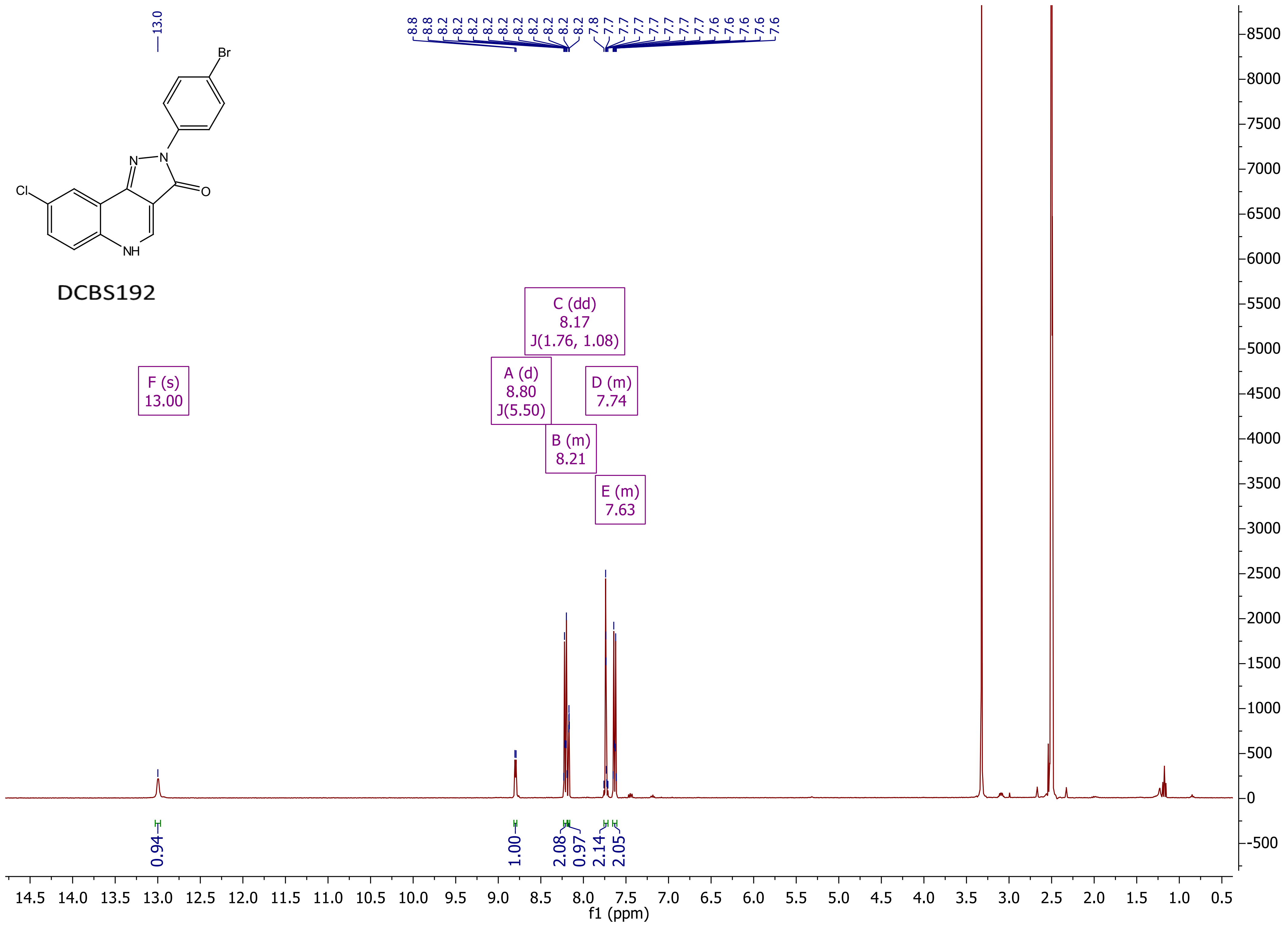

Br
N
N
O
Cl
NH
DCBS192
13.0
8.8
8.8
8.2
8.2
8.2
8.2
8.2
8.2
8.2
8.2
8.2
8.2
7.8
7.7
7.7
7.7
7.7
7.7
7.7
7.7
7.6
7.6
7.6
7.6
7.6
C (dd) 8.17 J(1.76, 1.08)
F (s) 13.00
A (d) 8.80 J(5.50)
D (m) 7.74
B (m) 8.21
E (m) 7.63
0.94
1.00
2.08
0.97
2.14
2.05
f1 (ppm)

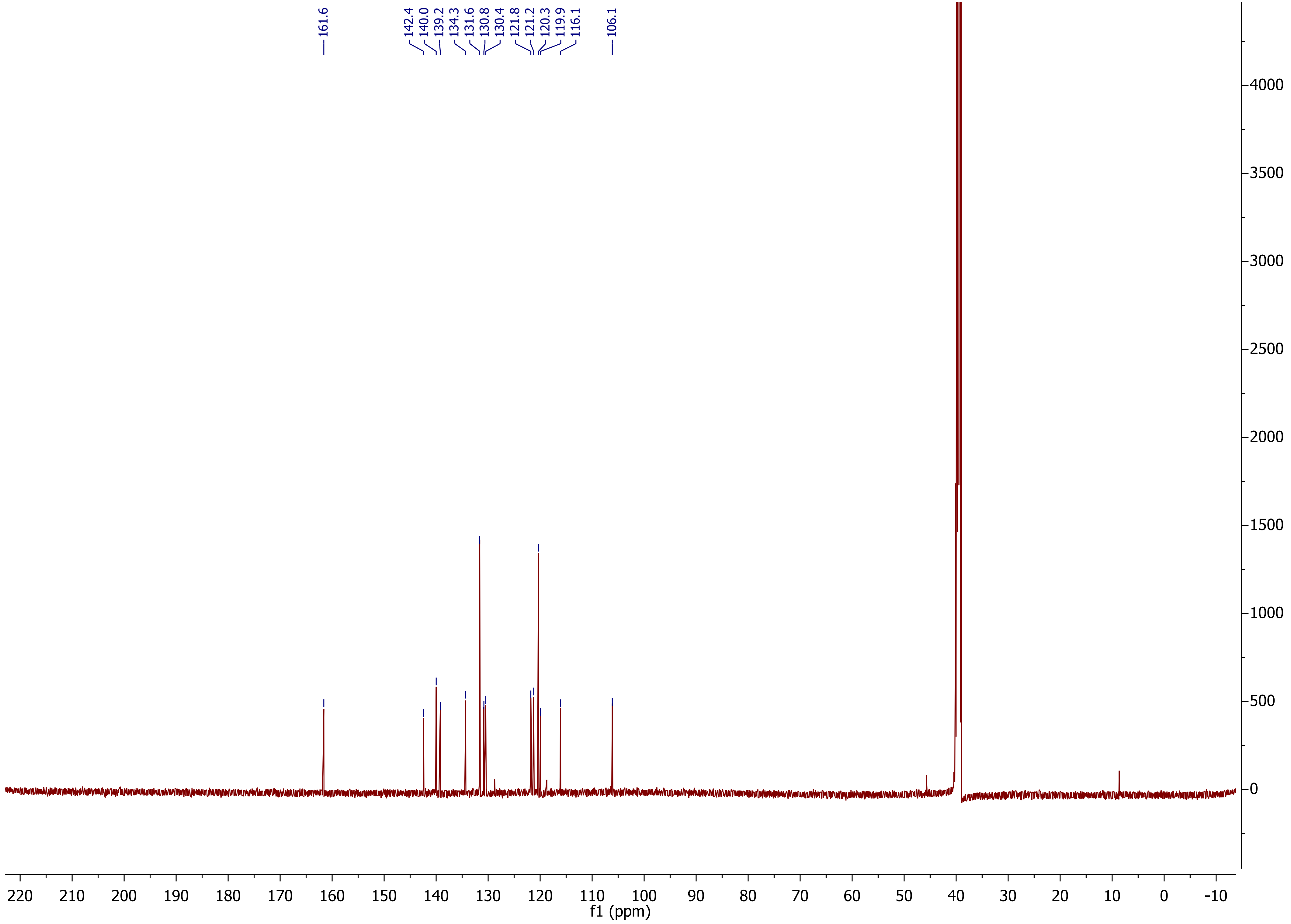

161.6
142.4
140.0
139.2
134.3
131.6
130.8
130.4
121.8
121.2
120.3
119.9
116.1
106.1
4000
3500
3000
2500
2000
1500
1000
500
0
220
210
200
190
180
170
160
150
140
130
120
110
100
90
80
70
60
50
40
30
20
10
0
-10
f1 (ppm)
